# Patient safety culture assessment in five public hospitals of Sichuan Province: a cross-sectional study with HSOPSC 2.0

**DOI:** 10.3389/fpubh.2025.1703929

**Published:** 2025-12-15

**Authors:** Wenjun Li, Chunyan Peng, Li Gu, Wenjing Fu, Fangmei Tang

**Affiliations:** 1 Department of Obstetric Nursing, West China Second University Hospital, Sichuan University, Chengdu, China; 2 Key Laboratory of Birth Defects and Related Diseases of Women and Children (Sichuan University), Ministry of Education, Chengdu, China

**Keywords:** patient safety culture, HSOPSC2.0, hospital, safety events, China

## Abstract

**Background:**

Patient safety is a core issue in the medical field, influenced by patient safety culture (PSC). Research on PSC in southwestern China remains insufficient.

**Methods:**

This study is a cross-sectional survey. A convenience sample of healthcare workers from five hospitals in Sichuan Province was surveyed during January to March 2025. The Chinese version of the Hospital Survey on Patient Safety Culture 2.0 was used to assess participants’ perceptions of PSC. SPSS 22.0 was used for data analysis, including descriptive statistics, Pearson correlation, *t*-tests, and ANOVA. Thematic analysis addressed open-ended comments.

**Results:**

This study collected 468 valid questionnaires. Among the participants, the majority were female (91.2%) and nurses (80.3%). The overall average positive response rate for PSC was 69.4%. Five dimensions were identified as strengths, with positive response rates ranging from 75.4 to 83.4%: “Communication about error,” “organizational learning-continuous improvement,” “team work,” “support for patient safety,” and “handoffs and information exchange.” In contrast, four dimensions required improvement (positive response rates: 51.5–71.2%), including “staffing and work pace,” “response to error,” “reporting patient safety events,” and “communication openness.” Notably, the positive response rate for “reporting patient safety events” was 53.1%, which was significantly lower than the 76.0% reported by AHRQ in 2024. Univariate analysis demonstrated statistically significant associations between safety culture scores and several variables, including working hours per week, work unit, adverse event reporting frequency, and self-reported overall patient safety ratings (*p* < 0.05). Qualitative analysis of open-ended comments identified seven primary themes, emphasizing the need for improvements in staffing levels, workload management, and administrative support.

**Conclusion:**

Our study reveals that the patient safety culture among healthcare professionals in Sichuan Province is at a moderate level, has not reached the expected standard. Notably, there exist significant gaps in critical domains, such as patient safety events reporting, when compared with developed regions, which underscores the imbalance in medical safety concepts across different regions. Future efforts should focus on establishing non-punitive reporting and learning mechanisms, optimize the allocation of human resources and clinical workflows, and ultimately foster a more robust and advanced PSC.

## 1 Background

Patient safety, as a fundamental concern in global healthcare, has emerged as a critical indicator for assessing healthcare quality. The World Health Organization (WHO) explicitly highlights in its *Global Action Plan for Patient Safety 2021–2030* that the increasing complexity of healthcare environments has contributed to a rising prevalence of patient safety risks associated with medical errors (1). Unsafe care is a major public health problem that affects millions of patients worldwide, with estimates suggesting that more than 1 in 10 patients suffer from adverse events. Unsafe care causes more than 3 million deaths every year globally, and that around half of all harm due to unsafe care is preventable (2). Patient safety events resulting from unsafe healthcare practices causes irreversible reputational damage to healthcare systems. It has an adverse effect on patient experience, trust in and engagement with healthcare services, the morale and well-being of healthcare workers, public perception of resource allocation to healthcare, and imposes a substantial economic burden (2). Therefore, healthcare systems must prioritize patient safety as a fundamental imperative.

A well-known cause of unsafe healthcare services is a weak patient safety culture (3). Patient safety culture (PSC) is defined as “An integrated pattern of individual and organizational behavior, based upon shared beliefs and values that continuously seeks to minimize patient harm, which may result from the processes of care delivery” (4). PSC is regarded as a barometer for measuring hospital management’s commitment to patient safety and the provision of high-quality healthcare services. Core components of PSC identified in previous research include leadership, teamwork, evidence-based, communication, learning, just, and patient-centered (5). In healthcare institutions, particularly hospitals, a positive PSC is primarily determined by communication based on mutual trust, effective information sharing, a shared understanding of the critical importance of internal security, organization learning, staff support and a non-punitive response to errors (6). In *To Err is Human: Building a Safer Health System*, the National Academy of Medicine (NAM, formerly the Institute of Medicine) highlighted that a ‘blame culture’ drives healthcare professionals to hide errors. The concealment of minor mistakes may result in serious harm, and organizations lose valuable opportunities to learn from these errors and make systemic improvements (3). A well-established PSC within healthcare institutions encourages professionals to report errors proactively, discuss risks openly, learn from mistakes, and implement improvements. This reduces the risk of harm at its source. This approach better meets patient needs, minimize adverse events and safeguards patient safety, and enhances the quality of healthcare delivery (7–9). Moreover, PSC is closely linked to the well-being of healthcare professionals. Several empirical studies have demonstrated an association between PSC and occupational burnout (10), workplace violence (11), job satisfaction and intention to leave the organization (12), thereby impacting the physical and mental health of healthcare staff and team stability. Therefore, developing and maintaining a robust PSC should be a strategic priority for any healthcare institution seeking sustainable improvements in patient care and safety.

The Agency for Healthcare Research and Quality (AHRQ) considers PSC assessment to be a vital part of improving healthcare quality (13). A comprehensive PSC assessment is essential for identifying both the strengths and weaknesses of it. It pinpoints areas for improvement and helps to identify systemic issues, monitor their progression, develop targeted interventions and implement change processes (14). In recent years, Chinese researchers have conducted studies on PSC across multiple regions and populations. However, the majority of these studies have focused on Taiwan and the eastern and central of China (15–19), with research in southwestern China being particularly scarce. Studies of PSC in multiple regions indicate that, despite variations in study districts, core vulnerabilities in PSC demonstrate consistency. These include deficiencies in non-punitive responses to errors, reporting patient safety events and staffing (17, 20–24). However, due to disparities in economic development, cultural practices, and policy support, PSC exhibit varying degrees of difference across regions and healthcare institutions. The southwestern region of China is characterized by relatively limited medical resources and demonstrates notable differences compared to eastern coastal areas in terms of collaboration models between primary healthcare institutions and central hospitals, as well as in the working environment for medical professionals. Therefore, more diverse evidence is necessary to support informed decision-making.

In the *Sichuan Province 14th Five-Year Plan for Health Development*, the People’s Government of Sichuan outlined strategic requirements to enhance medical quality and safety standards, strengthen the development of safe hospitals, and continuously improve the security prevention capabilities of medical institutions, emphasized the critical importance and urgency of elevating PSC within healthcare facilities (25). As the core of the Southwest Medical Consortium, the healthcare network spanning West China Hospital and West China Second University Hospital now covers the entire province of Sichuan and neighboring regions. Its distinctive strengths in PSC can be shared with primary care hospitals through technical collaboration and personnel training, but its weaknesses could also spread. To date, the only single-center survey on PSC conducted in Sichuan Province remains a 2012 study, which focused exclusively on nursing staff at West China Hospital of Sichuan University. Now over a decade old, this research excluded key healthcare groups such as physicians, medical technologists, and administrators (22), that gives rise to limitations in terms of temporal relevance, demographic coverage, and institutional representativeness. Therefore, we conducted a PSC survey across five public hospitals in Sichuan Province using the HSOPSC 2.0 scale. The primary objectives were to: (1) Systematically assess healthcare professionals’ perceptions of PSC levels in 5 hospitals in Sichuan; (2) Identify core weaknesses in patient safety culture development through comparative analysis with domestic and international data; (3) Provide evidence-based support for improving patient safety culture in Sichuan and the southwestern region, thereby promoting synergistic enhancement of patient safety and healthcare quality.

## 2 Methods

### 2.1 Study design

This study adopts a descriptive cross-sectional design, conducted among healthcare professionals across five medical institutions in Sichuan Province. These institutions comprise three general hospitals and two specialized hospitals (West China Hospital, West China Second University Hospital, along with their medical consortium hospital). In terms of hospital level, two are national, one is provincial, one is municipal and one is district and county. In terms of regional distribution, three hospitals are located in Chengdu, one in Ya’an and one in Zigong, covering northern, western and southern areas of Sichuan.

### 2.2 Samples

All participants were recruited using convenience sampling. We contacted administrators at five participating hospitals, who assisted in distributing recruitment notices. Individuals who voluntarily agreed to participate completed the survey via the Wenjuan Xing. The cover page of the electronic questionnaire included clear instructions indicating that participation was voluntary and anonymous, and that participants had the right to refuse or withdraw at any time. All collected data were securely stored in an encrypted folder, with personal information anonymized to ensure confidentiality.

*Inclusion criteria*: (1) Healthcare professionals working in public hospitals; (2) Age ≥18 years; (3) ≥1 year of continuous service in the current position at the institution; (4) Possession of basic comprehension abilities, with no communication barriers or mental health issues.

*Exclusion criteria*: (1) Non-hospital staff; (2) interns; (3) retired hospital staff; (4) individuals with cumulative leave exceeding 6 months within the past year.

*Calculation of questionnaire survey sample size*: The sample size is determined using the formula for exploratory research: $n=\frac{Z^{2}\cdot p\cdot (1-p)}{d^{2}}$ , *p* is the expected positive rate, *d* is the acceptable error, and *Z* is the confidence level. Our study primarily employs the Chinese version of the Hospital Patient Safety Culture Survey Version 2.0 (HSOPSC 2.0). Based on the 71% positive response rate reported by AHRQ in 2024 (26), we assume a positive response rate of 70% for our study, with *d* = 0.05 and *Z* = 1.96. The sample size calculation is as follows: *n* = 1.96^2^ × 0.7(1–0.7)/0.05^2^ = 322.69. Considering a 10% non-response rate, the estimated required sample size is: 323 ÷ (1–10%) = 359.

### 2.3 Instrument

The Chinese version of the HSOPSC 2.0 was used to conduct the questionnaire survey. This questionnaire was revised by AHRQ in 2019 based on the first edition. HSOPSC is an internationally recognized assessment tool for PSC, has been used extensively worldwide. HSOPSC 2.0 comprises 10 dimensions that comprehensively assess health professionals’ perceived PSC at both the individual and organizational levels (27). In 2023, WH Ying culturally adapted HSOPSC 2.0 into a Chinese version consisting of 9 dimensions, 32 items, 6 basic respondent information, and 2 additional items. These 2 additional items are: (1) the number of safety events reported by the respondent in the past 12 months (adjusted in our study to reflect the number of safety events reported by the respondent’s department); and (2) the perceived of Overall rating on patient safety (28). Most of the survey items use 5-point agreement scales (“Strongly disagree” to “Strongly agree”) or frequency scales (“Never” to “Always”) and also include a “Does not apply” or “Do not know” response option. The survey has a section at the end for open-ended comments. The questionnaire score ranges from 32 to 160, with the positive response rate serving as the primary evaluation metric. Positive response rate = Number of positive responses/(total number of respondents − number of missing responses).The number of positive responses refers to those who answered “Strongly Agree/Agree” and “Often/Always” (for reverse-scored items, it refers to those who answered “Strongly Disagree/Disagree” and “Never/Rarely”), the number of missing responses equals the sum of those who selected “Does not apply/Do not know” and those who provided no response. The positive response rate for each dimension is calculated based on the average positive response rate for all the items within that dimension. A positive response rate above 75% indicates an area of strength, while a rate below 50% indicates an area of weakness. A higher positive response rate signifies a stronger PSC. In the self-assessment of the overall patient safety rating, responses of ‘Very good’ or ‘Excellent’ are considered positive response (27). In Yin’s study, the content validity index (S-CVI) for the Chinese version of the HSOPSC 2.0 was 0.938. Item-level content validity indices (I-CVI) ranged from 0.833 to 1.000. Exploratory factor analysis showed that the nine dimensions explained 80.712% of the cumulative variance, and confirmatory factor analysis indicated a good model fit. The overall Cronbach’s *α* coefficient was 0.952, with Cronbach’s α for the nine dimensions ranging from 0.802 to 0.940. Test–retest reliability was 0.924, with dimension-specific reliability ranging from 0.710 to 0.905. Composite reliability (CR) ranged from 0.814 to 0.931 and average variance extracted (AVE) from 0.588 to 0.819. These findings suggest that the questionnaire is a reliable tool for assessing PSC in hospital settings (28). Prior to the formal data collection, we conducted a pre-test with eight healthcare professionals (five nurses, two doctors and a manager). By collecting feedback on the completion of the questionnaire and conducting one-to-one interviews to assess item comprehension, we did not identify any items that were ambiguously worded, semantically unclear or difficult to understand. Consequently, no modifications were made to the questionnaire. In our study, the Cronbach’s *α* coefficient for the Chinese version HSOPSC 2.0 was 0.916, with Cronbach’s *α* coefficients for the nine items ranging from 0.682 to 0.916.

### 2.4 Data collection

This survey was conducted between January and March 2025. We sent the survey link and QR code to hospital administrators, who then distributed them to their staff along with instructions on how to complete the survey. The questionnaire can be completed via mobile phone or computer. The homepage provides a detailed explanation of the objectives of our study, instructions for completing the questionnaire and a request for informed consent. The questionnaire is configured to allow only one submission per participant.

### 2.5 Data analysis

All data were imported into Excel 2019 for preliminary organization. Items within HSOPSC2.0 that were phrased in negative terms were reverse-coded so that higher scores indicated more positive responses. After determining the positive response rates for each item and dimension, missing data were imputed using multiple imputation methods. Statistical analysis was performed using SPSS 22.0. First, a P–P plot was used to examine the normality of data distribution, and Levene’s test was conducted to verify the homogeneity of variances. Data were confirmed to follow a normal or approximately normal distribution, and the variance homogeneity assumption was satisfied. Descriptive statistics are presented as percentages, means, and standard deviations. Differences in patient safety culture (PSC) across variables were examined using correlation analysis, independent samples *t*-tests, and one-way analysis of variance (ANOVA). For ANOVA results with statistical significance (P < 0.05), the LSD post-hoc test was further performed to identify specific pairwise differences between groups. A *p*-value of <0.05 was considered statistically significant.

Additionally, open-ended comments were subjected to qualitative content analysis, with thematic analysis adopted as the specific analytical approach. Thematic analysis followed a systematic procedure. First, a researcher with experience of qualitative research familiarized themselves with all the comments before conducting open coding of the raw data. Second, an initial coding framework was constructed based on the semantic meaning of the comments. Third, to ensure coding consistency, a second researcher reviewed the coded results to verify that the content aligned with the themes. Where discrepancies arose that could not be resolved through discussion, a nursing expert proficient in qualitative research was consulted to reach a consensus.

## 3 Results

### 3.1 Demographic and professional characteristics

A total of 468 valid questionnaires were collected. In terms of demographics, the average age of participants was 33.38 ± 6.82. Among them, 91.2% were female, 67.9% were married, and 87.6% held a bachelor’s degree or higher. Regarding professional backgrounds, the majority of participants (63.0%) worked in general hospitals, and the hospital level was relatively balanced across different hospitals. In terms of occupational roles, nurses accounted for the largest share (80.3%). By work unit, Obstetrics and Gynecology was the most common (39.0%), trailed by surgery (21.2%) and internal medicine (14.6%); other units constituted smaller proportions. For professional titles, junior and intermediate levels dominated, collectively representing 94.4%. Regarding working hours, most participants worked 41–50 h per week (66.9%). Furthermore, 93.4% of health professionals required direct patient contact in their work (see Table 1).

**Table 1:** Demographic and professional characteristics (N = 468).

Variables	Category	Frequency	Percent (%)/mean (SD)
Age			33.38 ± 6.82
Gender	Male	41	8.8
	Female	427	91.2
Marital status	Single	138	29.5
	Married	318	67.9
	Divorcee	12	2.6
Educational status	Junior college	58	12.4
	Bachelor	364	77.8
	Master or above	46	9.8
Hospital category	General hospital	295	63.0
	Specialized hospital	171	36.5
Hospital level	National level	127	27.1
	Provincial level	93	19.9
	Municipal level	155	33.1
	District and county-level	93	19.9
Staff position	Nurse	375	80.3
	Doctor	46	9.9
	Administrator	17	3.6
	Medical support staff	14	3.0
	Others	16	3.4
Work area at the hospital	Gynecology and obstetrics	182	39.0
	Surgery	99	21.2
	Internal medicine	68	14.6
	Pediatrics	37	7.9
	Outpatient department	26	5.6
	Other units	55	11.8
Professional title	Junior title	216	46.2
	Intermediate title	226	48.2
	Senior title	26	5.6
Hospital experience	<3 years	90	19.2
	3 ~ 5 years	81	17.3
	6 ~ 10 years	139	29.7
	>10 years	158	33.8
Unit experience	<3 years	54	11.5
	3 ~ 5 years	141	30.1
	6 ~ 10 years	139	29.7
	>10 years	134	28.6
Work hours per week	30 ~ 40	89	19.0
	41 ~ 50	313	66.9
	>50	66	14.1
Direct interaction with patients	Yes	436	93.4
	No	31	6.6

### 3.2 Patient safety culture status

*HSOPSC 2.0 scores and positive response rates*: The average HSPOSC 2.0 score among all participants was 123.01 ± 16.62, with an average positive response rate of 69.4%. Five dimensions achieved a positive response rate ≥75%, ranked from highest to lowest as follows: “Communication about error” (83.4%), “Organizational learning-continuous improvement” (80.4%), “Team work” (79.8%), “Support for patient safety” (78.0%) and “Handoffs and Information exchange” (75.4%). These represent areas of strength within the PSC. The positive response rates across the four dimensions ranged from 50 to 75%. The lowest rates were recorded for “Staffing and work pace” (51.5%), “Response to error” (51.9%), “Reporting patient safety events” (53.1%) and “Communication openness” (71.2%). While no dimension was classified as a disadvantageous area (Table 2), these areas require improvement.

**Table 2:** The score and response situation of the hospital safety culture (*N* = 468).

Patient safety culture dimensions	Mean (SD)	Positive response (%)	Neutral response (%)	Negative response (%)
Composite measure average	123.01 ± 16.62	69.4	17.4	13.2
Team work	4.03 ± 0.69	79.8	8.9	11.3
Staffing and work pace	3.35 ± 0.79	**51.5**	23.4	25.1
Organizational learning-continuous improvement	4.03 ± 0.64	80.4	11.9	7.7
Response to error	3.47 ± 0.80	**51.9**	25.4	22.7
Support for patient safety	3.98 ± 0.62	78.0	14.8	7.2
Communication about error	4.32 ± 0.73	83.4	12.9	3.7
Communication openness	3.98 ± 0.67	**71.2**	19.5	9.3
Handoffs and information exchange	3.88 ± 0.74	75.4	14.5	10.1
Reporting patient safety events	3.56 ± 1.06	**53.1**	25.7	21.2

The positive response rate of each dimension = the average of the sum of the positive rating percentages of each item. Key Laboratory of Birth Defects and Related Diseases of Women and Children (Sichuan University), Ministry of Education. Bolded values: Lower than the target value (positive response rate≥75%).

*Number of patient safety events reported*: 25.1% of healthcare professionals indicated that no patient safety event had been reported in their unit in the last 12 months. The largest proportion (35.5%) reported one or two events, followed by three to five events (24.4%). The lowest proportion (5.8%) reported 11 or more (Table 3).

**Table 3:** The status of adverse event reporting and self-assessed patient safety (*N* = 468).

Variables	Category	Frequency	Percent (%)
Number of patient safety events reported among 12 months in the unit	None	118	25.3
	1 ~ 2	166	35.5
	3 ~ 5	114	24.4
	6 ~ 10	42	9.0
	≥11	27	5.8
Overall rating on patient safety	Bad	0	0
	Average	87	18.6
	Good	142	30.4
	Very good	146	31.3
	Excellent	92	19.7

*Overall rating on patient safety*: no healthcare professional rated the patient safety level in their department as “Bad,” 51% gave positive responses about the overall patient safety level in their unit, rating it as “Excellent” or “Very Good” (Table 3).

**Figure 1:**
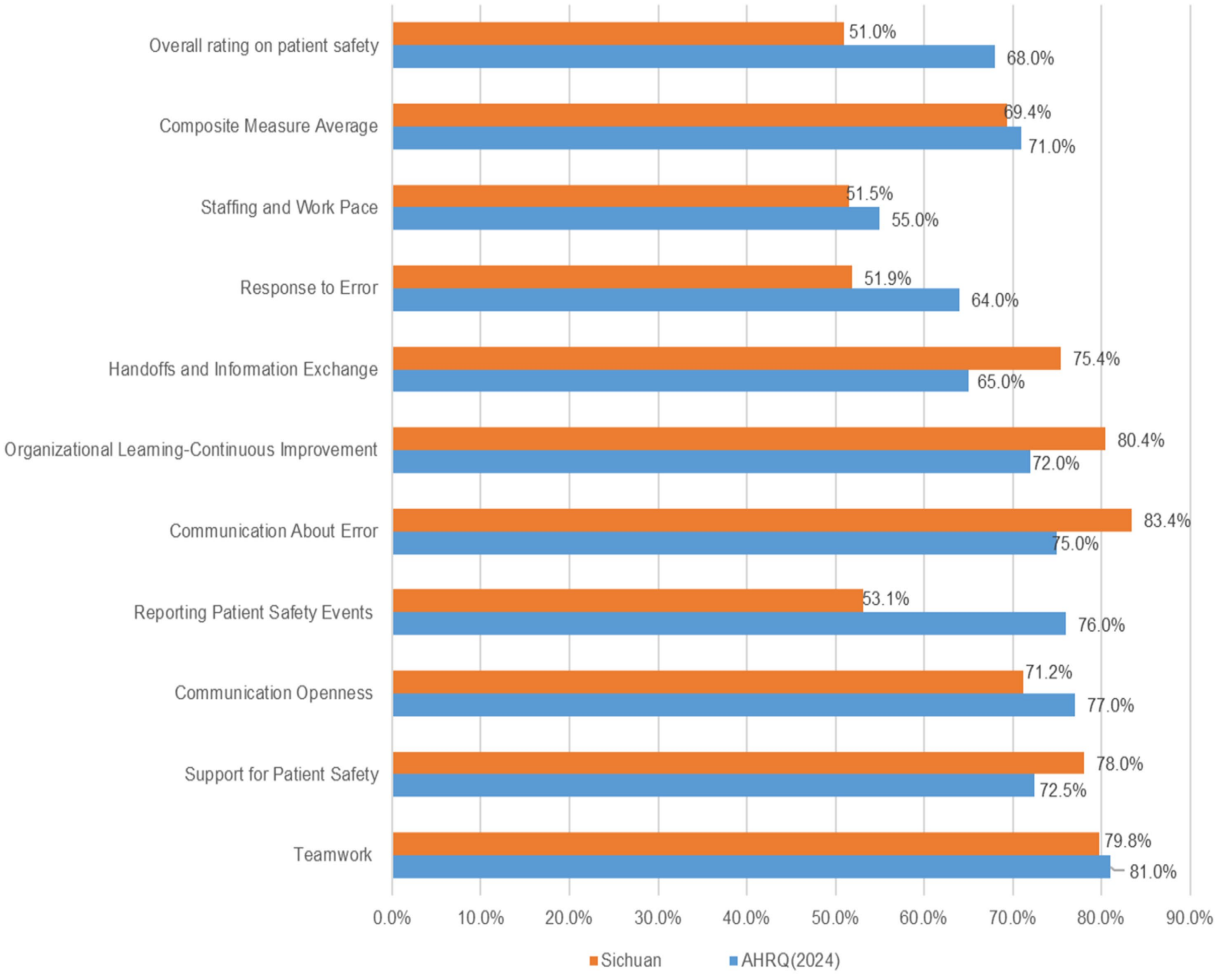
Comparative results for patient safety culture composite measures.

We compared our findings with the 2024 data of the United States (U.S.) published by AHRQ (26) (Figure 1). The results showed that the positive response rate for Sichuan Province’s PSC was slightly lower than that of the U.S. (69.4% vs. 71%). Among the nine dimensions, five scored below the U.S. benchmarks, while four were higher. The dimension on “Reporting patient safety events” exhibited the largest discrepancy compared to the AHRQ data (53.1% vs. 76.0%). Additionally, the proportion of positive self-assessments regarding patient safety levels also fell behind it (51.0% vs. 68.0%).

Additionally, we gathered 18 suggestions from participants regarding improvements to patient safety through the open-ended questions in our questionnaire. Through thematic analysis, we identified seven themes, each with specific sub-themes reflecting key concerns and proposed measures. They are “Insufficient staffing and overwork,” “Excessive non-clinical burdens and lack of rest protection,” “Insufficient team management and personnel support,” “Insufficient management fairness affects clinical decision-making,” “Medical environment and resource constraints,” “Need for safety training and process standardization,” and “Personalized patient care management and health education” (Table 4). These themes reflect healthcare professionals’ perspectives on systemic, environmental, and operational factors influencing patient safety, as well as actionable strategies to address these challenges. The findings mainly related to the core dimensions of PSC: “Support for patient safety” (four themes, nine comments and recommendations) and “Staffing and work pace” (two themes, four comments and recommendations).

**Table 4:** Suggestions and opinions of medical staff on patient safety.

Theme	Theme description	Subthemes and statements	Corresponding to the PSC dimensions
Insufficient staffing and overwork	This topic focuses on the issue of an imbalance between the number of healthcare workers and their workload, and it is a core demand that is frequently mentioned.	Subtheme 1: Insufficient staffing of medical staff: “Strongly urge for an increase in staff,” “Provide sufficient nursing personnel,” “There are too many patients, but few staff.” Subtheme 2: Excessive workload affects work quality: “Clinical work is too busy,” “The work is too hectic and saturated,” “When the workload is too large, personnel cannot be adequately equipped, resulting in the inability to complete all tasks with quality and quantity.”	Staffing and work pace
Excessive non-clinical burdens and lack of rest protection	This theme emphasizes the impact of excessive non-clinical tasks and the infringement of rest time on work status.	Subtheme 1: Heavy non-clinical tasks: “The hospital has way too many trainings, too many rules, and really tough promotion requirements. Doctors cannot possibly focus all their energy on serving patients.” Subtheme 2: Lack of protection for the right to rest: “Our rest times are always being taken up. We hope our leaders can schedule shifts reasonably, give us back our normal rest time. just let us catch a breath.”	Staffing and work pace Support for patient safety
Insufficient team management and personnel support	This theme involves issues regarding team stability, incentive mechanisms, and the protection of medical staff’s own rights and interests.	Subtheme 1: Insufficient team stability and recognition: “Our team is unstable, there is no reasonable performance distribution mechanism or incentive system, and both hospital leaders and patients ignore the efforts of the nursing team.” Subtheme 2: Demands for rights protection and interests: “It is necessary to strengthen the protection for medical personnel, including their salaries, personal safety protection, and so on.”	Support for patient safety
Insufficient management fairness affects clinical decision-making	This theme mainly points to the potential negative impact of management behaviors on clinical work.	Subtheme: Leadership behavior affecting decision-making fairness: “Excessive care for acquaintances by leaders can lead to deviations in clinical decision-making.”	/
Medical environment and resource constraints	The central focus of this theme is the examination of how patient safety is constrained by both the hardware environment and resource allocation.	Subtheme: Space and facilities issues: “It is suggested that fewer extra beds be added. How can patient safety be ensured if too many extra beds are added?,” “Our hospital wards are too old, with many facilities aging, and such an environment has potential safety risks.”	Support for patient safety
Need for safety training and process standardization	It is emphasized that patient safety should be improved via training and standardized processes.	Subtheme 1: Demands for specialized training: “We hope that hospitals can attach greater importance to and strengthen the occupational safety protection and patient safety training for interns.” Subtheme 2: Requirements for process standardization: “In order to ensure patient safety, healthcare workers must follow established procedures without exception.”	Support for patient safety
Personalized patient care management and health education	This theme proposes targeted measures to enhance safety from the patient’s perspective.	Subtheme 1: Individualized treatment for patients: “Different interventions should be employed according to patients’ specific circumstances, as targeted practices could improve patient safety.” Subtheme 2: Strengthening health education: “We should provide more health education to patients to ensure their safety.”	/

### 3.3 Differences in health professional’s perceptions of patient safety culture

Correlation analysis revealed no significant linear relationship between age and PSC scores (*p* > 0.05). Independent-samples *t*-tests ANOVA results showed no statistically significant differences in PSC among healthcare professionals categorized by gender, marital status, hospital category, hospital level, professional role, years of work experience, professional title, or direct patient contact status (*p* > 0.05, Table 5).

**Table 5:** The differences in the patient safety culture among different variables (*N* = 468).

Variables	Category	Mean (SD)	Statistical analysis	*p-*value
Age			*r* = 0.064	0.170
Gender	Male	119.82 ± 15.93	*t* = 1.288	0.198
	Female	123.32 ± 16.67		
Marital status	Single	122.93 ± 16.57	*F* = 2.366	0.950
	Married	123.44 ± 16.50		
	Divorcee	112.84 ± 18.61		
Educational status	Junior college	124.21 ± 16.50	*F* = 0.722	0.486
	Bachelor	123.15 ± 16.55		
	Master or above	120.42 ± 17.44		
Hospital category	General hospital	122.45 ± 17.83	*t* = −1.013	0.312
	Specialized hospital	123.99 ± 14.35		
Hospital level	National level	122.24 ± 17.04	*F* = 2.020	0.110
	Provincial level	119.85 ± 15.26		
	Municipal level	124.87 ± 17.59		
	District and county-level	124.16 ± 15.39		
Professional	Nurse	123.42 ± 16.83	*F* = 0.735	0.568
	Doctor	121.87 ± 15.75		
	Administrator	124.25 ± 16.19		
	Medical support staff	120.64 ± 13.66		
	others	116.78 ± 17.46		
Workplace at the hospital	Gynecology and obstetrics^a^	126.72 ± 16.43**bc*f	*F* = 4.517	<0.001
	Surgery^b^	119.23 ± 17.06**a*e		
	Internal medicine^c^	118.63 ± 15.85**a*e		
	Pediatrics^d^	124.62 ± 16.54		
	Outpatient department^e^	126.39 ± 15.45*b*c		
	Other units^f^	120.32 ± 15.36	*F* = 0.154	0.857
Professional title	Junior title	123.26 ± 17.03		
	Intermediate title	122.64 ± 16.45		
	Senior title	124.26 ± 15.15		
Hospital experience	<3 years	123.33 ± 15.78	*F* = 1.145	0.330
	3 ~ 5 years	119.90 ± 16.62		
	6 ~ 10 years	123.67 ± 18.35		
	>10 years	123.01 ± 16.62		
Unit experience	<3 years	121.62 ± 16.61	*F* = 0.380	0.768
	3 ~ 5 years	122.23 ± 16.11		
	6 ~ 10 years	123.72 ± 18.32		
	>10 years	123.66 ± 15.38		
Work hours per week	30 ~ 40^a^	121.58 ± 17.70	*F* = 3.159	0.043
	41 ~ 50^b^	124.27 ± 16.37*c		
	>50^c^	119.02 ± 15.77*b		
Direct interaction with patients	No	119.58 ± 16.09	*t* = −1.190	0.235
	Yes	123.26 ± 16.65		
Number of adverse event reports among 12 months	None^a^	120.73 ± 17.32*ce	*F* = 3.620	0.006
	1 ~ 2^b^	120.76 ± 16.32*ce		
	3 ~ 5^c^	126.74 ± 16.69*ab		
	6 ~ 10^d^	124.86 ± 15.00		
	≥11^e^	128.24 ± 14.03*ab		
Self-assessed patient safety level	Average^a^	109.61 ± 12.87**bcd	*F* = 51.727	<0.001
	Good^b^	119.76 ± 13.60**acd		
	Very good^c^	126.59 ± 14.17**abd		
	Excellent^d^	135.05 ± 17.22**abc		

a, In the LSD post hoc test, compared with a, the difference is statistically significant; ab, In the LSD post hoc test, compared with a and b, the difference is statistically significant. * *p* < 0.05. ** *p* < 0.001.

ANOVA results revealed statistically significant differences in PSC scores among health professionals in different work units (*F* = 4.517, *p* < 0.001). Scores in the Obstetrics and Gynecology Outpatient Department were significantly higher than those in the Surgery and Internal Medicine category. Statistically significant differences in PSC scores were observed among health professionals with different working hours (*F* = 3.159, *p* = 0.043): those working 41–50 h per week scored significantly higher than those working over 50 h and less than 40 h per week. Scores in the three to five and ≥11 patient safety event report groups were significantly higher than those in the no reports and one to two reports groups. There were statistically significant differences in PSC scores across different self-assessed levels of patient safety (*F* = 51.727, *p* < 0.001), with scores showing a marked upward trend as self-assessment levels increased (see Table 5).

## 4 Discussion

This is the first time the HSOPSC 2.0 has been used to assess PSC in Sichuan Province, China. We analyzed the perceived PSC levels among 468 healthcare professionals using a questionnaire survey to identify areas for improvement. This aims to provide evidence-based support for optimizing patient safety management systems in hospitals. The results indicate that the average positive response rate for the PSC among healthcare professionals is 69.4%, which has not yet reached the target of 75%. This is consistent with the outcomes of a nationwide survey in China conducted by Huang et al. (24) between 2020 and 2021, using the HSOPSC 1.0, the study surveyed 8,164 healthcare professionals and reported a positive response rate of 69.68%. Additionally, the rate in the current study is higher than those documented in South Korea (43%) (29), Malaysia (53.1%) (30), and Saudi Arabia (62%) (31). However, it is slightly lower than the 2024 AHRQ data (71%) (26), Brazil (70.6%) (32), and a survey of 9 private hospitals and 11 clinics in China (76%) (19). 51% of participants rated the patient safety level of their unit as “very good” or “excellent,” which is lower than the 68% positive rating in the U.S. (26). Data comparisons indicate that Sichuan Province has achieved some success in establishing a PSC, but more effort is needed. Differences in PSC across countries may be associated with various factors, such as cultural background, the characteristics of healthcare systems and the level of emphasis placed on patient safety. These factors interact with each other. Compared with developed countries such as U.S., Sichuan Province may have gaps in healthcare resource allocation and hospital management philosophies. The AHRQ long-term advancement of patient safety management and continuous improvement mechanisms may be positively correlated with the country’s comparatively high PSC response rate. Data from private hospitals in China exceeds that from Sichuan Province, which is potentially attributable to operational models and volumes. Private hospitals in China are usually smaller than public hospitals and tend to treat patients with less severe conditions. Furthermore, as profit-driven entities, private hospitals often prioritize patient experience, viewing PSC as a key competitive advantage. Their management is more flexible, enabling them to respond swiftly to issues arising in safety culture development and resolve them.

Among the PSC dimensions, the lowest positive response rates were observed in the areas of “Staffing and work pace,” “Response to error” and “Reporting patient safety events,” at 51.5, 51.9 and 53.1%, respectively. These findings are consistent with the results of a survey conducted across 26 provinces in China, in which positive response rates for the dimensions “Non-punitive responses to errors,” “Staffing” and “Frequency of events reported” were found to be below the control limits, ranging from 43.4 to 52.8% (24). When compared with the AHRQ data, our study reveals both similarities and differences. The similarity lies in the domains of “Staffing and work pace” and “Response to error” with the lowest positive response rates, at 55.0 and 64.0% respectively. The divergence lies in the domain of “Reporting patient safety events,” a dimension where US hospitals demonstrate a strength in PSC, achieving a positive response rate of 76% (26). Notably, these discrepancies align with previous findings from PSC surveys in eastern China. Study by Lin et al. (23) of five general hospitals and eight specialized hospitals in Shenzhen revealed that healthcare professionals’ positive response rates in the “Staffing” and “Nonpunitive responses to errors” domains both <50%, indicating urgent need for improvement. However, the positive response rate in the “Frequency of events reported” domain reached 72% (23), significantly higher than the findings of our study. There may be several reasons for this difference, one of which could be policy orientation. The United States has a non-punitive events reporting culture that is focused on systemic improvement, which is centered on the principle that most patient safety events stem from systemic vulnerabilities rather than individual errors. Reporters are explicitly exempt from liability under this system. Shenzhen has established a comprehensive regulatory framework underpinned by legislation such as the *Shenzhen Special Economic Zone Medical Regulations*. This framework covers everything from institutional self-assessment to departmental oversight. Its safety culture emphasizes non-punitive measures to encourage proactive reporting, maintaining accountability through clearly defined reporting obligations and procedural requirements. In contrast, Sichuan’s culture is dominated by administrative oversight and post-incident rectification. Although it also encourages reporting of safety events, the prevailing culture places greater emphasis on enforcing standards through administrative intervention and retrospective accountability.

Globally, staffing and non-punitive responses to errors are key issues in achieving a PSC (15, 16, 23, 24, 26, 33). Staffing shortages may be driven by multiple factors. First, the mismatch between rising healthcare demand and insufficient supply of healthcare workers is a common challenge across both developed and developing countries (34). On the one hand, healthcare professionals require an extended training period; on the other, high occupational stress contributes to talent attrition. These two issues together make it difficult for the number of healthcare workers to keep pace with the growing patient demand. This directly results in a situation where staffing cannot meet the workload. Moreover, issues such as inequitable distribution of resources, inefficient workflows and excessive non-clinical tasks persist both across different levels of hospitals and within individual institutions, further exacerbating the perception of a hectic work pace. Additionally, some hospitals lack effective fatigue management mechanisms. This combination of factors keeps healthcare workers in a perpetual state of high-intensity, high-stress labor, leading to lower positive response of “staffing and work pace.” The themes “Insufficient staffing and overwork” and “Excessive non-clinical burdens and lack of rest protection” extracted from open comments, further validate the authenticity and urgency of the aforementioned issues. In response to this situation, optimizing and implementing the tiered diagnosis and treatment system, streamlining workflows based on clinical needs, adopting scientific scheduling practices, and reducing non-essential non-clinical tasks may prove to be effective solutions.

It should be noted that the interpretation of these differences in our study is based on an analysis of the correlations between the existing research. This interpretation has not been validated through empirical research to establish causality. Differences in PSC across regions and countries result from the interplay of multiple factors, including healthcare systems, sociocultural contexts and policy environments. Attributing these differences to a single dimension is inherently limiting, and further validation through subsequent cohort studies or interventional research is required to draw relevant conclusions.

A punishment-oriented responses to errors persists to varying degrees in healthcare institutions worldwide, while non-punitive responses remain underemphasized (35). In China, a blame culture continues to prevail, as validated by our findings. Medical errors are often directly linked to the performance evaluations and reputations of healthcare workers (36). In such a context, healthcare workers lack sufficient confidence in the fairness and supportive nature of error management systems. This leads them to conceal errors rather than proactively reporting them and participating in subsequent analysis. This climate severely hinders the implementation of the patient safety philosophy of learning from mistakes rather than blaming. In fact, punitive responses to errors not only fail to enhance patient safety but also obscure the potential causes of mistakes. This hinders the entire medical industry from learning from its mistakes. As the *Swiss Cheese Model* shows, when errors occur, the entire system and higher-level systems should be examined first, rather than blaming individuals (37). According to our study, the positive response rate for the “Communication about error” dimension was the highest, reaching 83.4%. However, the positive response rate for the “Communication Openness” dimension was below standard at 71.2%. This discrepancy may indicate that while hospital managers demonstrate proactive attention to safety events and a willingness to communicate about them, this focus has not translated into an open communication environment. Medical staff still lack the initiative to proactively discuss potential safety hazards within their units. It also confirms the deep-seated impact of a blame-oriented culture: when errors occur, hospital managers often attribute responsibility to individual incompetence rather than analyzing organizational deficiencies. This results in healthcare workers fearing punitive consequences and being discouraged from providing positive, proactive responses to safety hazards and incidents. The result is an awkward situation of “communication in form, but not in openness.” In terms of patient safety event reporting, U.S. and Eastern China significantly outperformed than the region covered by this study, with positive response rate of 76% (26) and 72% (23), respectively, compared to only 53.1% in our study. This disparity may be closely linked to a range of factors, including regional economic and healthcare development levels, the effectiveness of system implementation, and the extent of localization and adaptation. The U.S. healthcare system is well-established and has prioritized PSC for decades. It has not only established a non-punitive reporting system, but also cultivated a culture that encourages proactive reporting through ongoing guidance. In such an environment, healthcare providers do not need to fear being held accountable for reporting errors, which significantly increases their willingness and motivation to report incidents voluntarily. As a developed coastal city in eastern China, Shenzhen has achieved a leading position in the development of its healthcare sector, supported by a robust regional economy. It adopted advanced international medical safety management concepts and practices earlier than other cities, establishing a far more systematic and in-depth safety culture than less developed areas. This foundation enables Shenzhen to demonstrate superior performance in the critical area of patient safety incident reporting. Therefore, changing the way the healthcare system perceives and responds to errors is now an urgent priority. We must establish a learning-oriented error management mechanism at an organizational level. Instead of viewing errors as grounds for individual accountability, they should be treated as critical opportunities to optimize systems and enhance safety. This can be achieved by refining non-punitive patient safety event reporting systems, strengthening systemic root cause analysis and fostering open communication. This approach will ultimately lead to continuous improvement in healthcare safety standards.

Furthermore, an analysis of 18 patient safety-related comments revealed that the themes centered on two dimensions of PSC. “Patient Safety Support” and “Staffing and Work Pace,” and not all of them referred to disadvantage domains. This discrepancy was probably due to the differing measurement logics of the research tools. The HSOPSC 2.0 scale used standardized dimension scoring to focus on the overall perception of safety culture, with scores based on agreement with the current situation. In contrast, the qualitative open-ended questions encouraged spontaneous expression, focusing on the most urgent practical concerns for healthcare staff. These questions prioritized feedback on the most impactful and urgent issues requiring resolution rather than balanced evaluations across all dimensions. Together, they present a comprehensive picture of patient safety culture from distinct perspectives. Therefore, even if ‘Patient Safety Support’ is not categorized as a deficient dimension in quantitative research, managers and relevant departments must still pay full attention to it, addressing core issues closely tied to staff interests and patient safety.

We also found that the perceived PSC of health professionals differed depending on their workload. In our study, over 80% of participants reported working more than 40 h per week. Those working between 41 and 50 h per week perceived the highest level of PSC, significantly higher than those working over 50 h or less than 40 h per week (Table 5). This finding is consistent with a previous study conducted in Saudi Arabia (38). A moderate workload is more conducive to maintaining the professional vigilance of healthcare professionals. A workload that is excessively light may blunt their awareness of safety risks, whereas an overly heavy workload tends to trigger safety incidents and induce severe fatigue (39). By contrast, a moderate workload not only fulfills clinical needs but also mitigates excessive fatigue, allowing healthcare professionals to maintain a proactive perception of PSC. This could inform hospital scheduling: During staffing shortages, the workloads of healthcare professionals can be increased moderately, but long-term overburdening should be avoided.

Additionally, we found that health professionals in different work units perceive varying levels of PSC. Compared to those working in obstetrics and gynecology, health professionals in internal medicine and surgery report lower levels of PSC (Table 5). According to the characteristics of the clinical setting, the workloads and uncertainty in internal medicine and surgery environments are significantly higher (40), whereas the workflow in obstetrics and gynecology is more cyclical and predictable. Standardized procedures for pregnancy check-ups and established protocols for delivery care significantly reduce decision uncertainty for health professionals. Furthermore, obstetrics and gynecology safety protocols combine strong operational feasibility with high repeatability, mitigating the weakening of PSC perception caused by disorderly work pressures. Crucially, obstetrics primarily serves healthy mothers and newborns, a population with higher demands for medical safety. Safety events in this setting not only threaten maternal and infant health, but also trigger widespread societal concern, the impact of which far exceeds that of routine safety incidents in internal medicine and surgery (41). The significant safety and societal implications of safety events have fostered a greater awareness of safety risks among obstetric and gynecological health professionals in their daily work. This has led to a more proactive approach to patient safety requirements and a greater awareness of safety culture. Furthermore, the results of the ANOVA validate the positive practical value of PSC. The higher the level of PSC perceived by healthcare personnel, the more adverse events are reported and the higher their self-assessment of the patient safety level in their units (see Table 5). This finding is consistent with the key elements of PSC. Mature PSC emphasizes a non-punitive reporting environment, a continuous improvement mindset and shared ownership of safety responsibilities across all staff. Higher perceived PSC levels encourage healthcare professionals to proactively disclose potential safety risks and address adverse events. Concurrently, trust in the unit’s safety management system fosters more positive safety rating assessments – a direct manifestation of the effective implementation of PSC.

Our study’s contribution lies in its application of the Chinese version of the HSOPSC 2.0 to investigate PSC in Southwest China for the first time, thereby providing supplementary multidimensional value to existing research in this field. We present an overview of the overall PSC levels and dimensional distribution characteristics among healthcare professionals in five hospitals of varying levels and types in Sichuan Province. We identified critical weakness dimensions, thereby filling a research gap in standardized PSC data within Southwest China. This provides locally relevant reference points for the formulation of subsequent regional patient safety management policies. Furthermore, cross-regional and cross-national comparisons of Sichuan data with AHRQ data and coastal Chinese regions reveal common patterns and regional variations in PSC development across different healthcare systems and cultural contexts. This enriches the comparative evidence base for cross-cultural PSC research, offering new empirical support for understanding the distinct characteristics and developmental pathways of PSC within China’s western healthcare environment.

### 4.1 Limitations of the study

Although our study has several advantages, it also has limitations. First, our study selected the hospitals and population primarily based on the principle of data availability, employing convenience sampling to obtain the data, which may result in selection bias. While hospitals at different levels and in different regions were included to increase sample size, stratified or random sampling methods were not employed to improve representativeness. This may lead to selection bias, thereby affecting the generalizability of the findings. Second, the participant demographic exhibited a notable skew, the majority were nurses, with an overwhelming predominance of female respondents. In contrast, other healthcare professionals and hospital administrators were significantly underrepresented. The unbalanced composition of the sample may limit the generalizability of the research findings. This sample cannot reflect the perceptions of patient safety culture across the entire healthcare workforce, particularly as it struggles to encompass the views of healthcare professionals beyond the nursing cohort. Consequently, caution should be exercised when interpreting these results as indicative of the overall patient safety culture among healthcare personnel in Sichuan Province, or when using them to inform policy. However, from the perspective of the study’s core objectives, the perceptions of patient safety culture held by nurses, as the direct implementers of medical safety practices, are highly relevant to the central research questions and exert a critical influence. Therefore, the relatively high proportion of nursing staff in the sample may be rational and specific for accurately measuring and reasonably interpreting the core dimensions of patient safety culture. Third, this study may be subject to information bias, particularly self-report and social desirability bias, which could affect the validity of the findings. Data were primarily collected via self-report questionnaires, in which participants may have provided responses that aligned with social expectations or organizational norms rather than their true opinions. This could lead to an overestimation of positive patient safety support dimensions and an underestimation of problematic aspects, thereby distorting the actual situation. Furthermore, self-reported data is inherently subjective, as participants may interpret questionnaire items differently. Recall bias also cannot be ruled out, particularly for questions concerning past experiences or behaviors. These biases may have affected the accuracy of the quantitative results and the reliability of the conclusions drawn. Furthermore, the cross-sectional design of this study limits our ability to explore changes in PSC over time and makes it difficult to establish causal relationships between potential influencing factors and PSC outcomes. Fourth the study’s data sources were limited, relying on questionnaire surveys with only a small number of open-ended comments. This hindered the exploration of multidimensional underlying issues in PSC and the identification of potential hidden factors affecting it. This limited our ability to develop a comprehensive understanding of patient safety. To address these limitations, future research will proceed along the following lines: (1) Future research could use stratified sampling methods to include a more representative sample of hospitals and study populations. This would help to validate the perceptions of healthcare professionals regarding PSC and improve the generalizability and practical applicability of the findings. (2) According to the weaknesses in PSC, the future research could conduct qualitative research to complement existing quantitative findings. This would enable a deeper analysis of the root causes of these weaknesses and lead to a more comprehensive and systematic understanding of the current state of PSC. (3) Future research may employ study designs that are more conducive to causal inference, such as cohort or longitudinal tracking studies, to establish causal relationships between PSC and the safety behaviors of healthcare professionals. The dynamic characteristics and influencing factors of PSC levels across different time periods should also be systematically investigated.

## 5 Conclusion

Health professionals in Sichuan Province perceive PSC levels as moderate to above average; however, these figures still fall short of the desired targets. Significant gaps persist in some critical areas compared to developed nations and other developed regions of China. “Staffing and work pace,” “response to error” and “reporting patient safety events” are core areas requiring improvement, aligning with common challenges faced by healthcare institutions globally. Of particular concern is Sichuan’s performance in safety event reporting, which is significantly lower than that of U.S. and eastern coastal regions of China. This highlights uneven development in medical safety management across regions and underscores the need for non-punitive error reporting and organizational learning mechanisms at the systemic level, as well as optimizing human resource allocation and workflows. Furthermore, variations in perceptions of the PSC across different groups and units should be acknowledged, with a focus on addressing shortcomings and optimizing the system. In addition to addressing areas of weakness in the PSC, it is essential to identify and resolve core issues that are directly linked to the practical needs of healthcare staff and that have a profound impact on patient safety, in order to enhance the PSC comprehensively.

## Data Availability

The original contributions presented in the study are included in the article/supplementary material, further inquiries can be directed to the corresponding authors.
